# *De Rerum Natura*: How Do Halide Perovskites Self‐Heal From Damage?

**DOI:** 10.1002/adma.202518808

**Published:** 2026-03-17

**Authors:** Davide Raffaele Ceratti, Gary Hodes, David Cahen

**Affiliations:** ^1^ CNRS UMR 8247 IRCP Institut de Recherche de Chimie Paris Paris France; ^2^ Weizmann Institute of Science Rehovot Israel

**Keywords:** defects, halide perovskites, self‐healing, solar‐cells, stability

## Abstract

By actually addressing the title question, we provide a comprehensive and critical review of self‐healing (SH) in lead‐based halide perovskites (HaPs), a phenomenon with profound implications for the stability of these materials across all applications, from photovoltaics to light emission and radiation detection. We emphasize reasoning as a guide to interpreting the dynamic balance between degradation and recovery when HaPs are exposed to light, heat, mechanical stress, or radiation. We compile and assess what are, in our view, the most relevant, available reports of damage–healing dynamics, distinguishing verified facts and observations from interpretations and unresolved questions. Key topics include damage accumulation, light soaking, and photo‐brightening, as well as the mechanistic roles of lattice dynamics, halide migration, redox chemistry, and acid–base equilibria in the disappearance of defects on accessible time scales. Thus, we go beyond a conventional summary by providing a unifying framework to clarify contradictions in the literature and reveal the underlying principles of reversible damage. By consolidating results that are often scattered into a coherent picture, we strive to establish a foundation for predictive models of SH kinetics, while guiding strategies to stabilize devices. We anticipate that this critical synthesis will serve as an authoritative reference for the metal halide perovskite research field.

## Graphical abstract

How can lead‐halide‐perovskites self‐heal after damage from light, heat, mechanical strain, or radiation? We show that reversible chemical defects underly performance losses and recovery, highlighting the roles of halide and proton migration, redox‐driven reactions, and acid‐base equilibria. Unless material is lost to the environment, these processes will re‐form broken bonds, thus providing guidance for improving device stability.

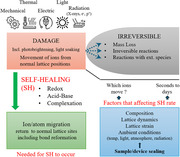

## 1 Introduction

**FIGURE 1:**
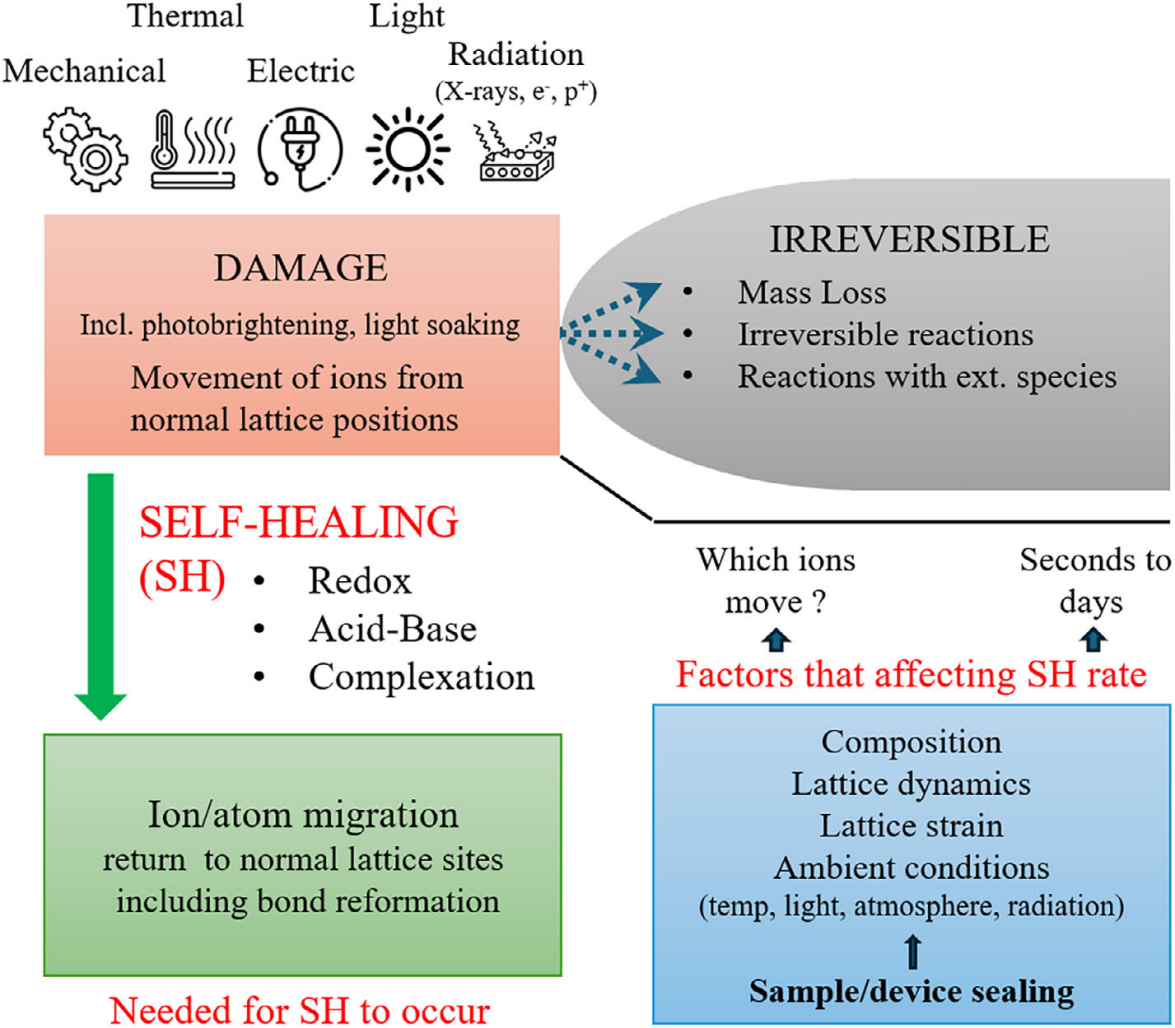
Scheme of the concepts treated in this work.

This review aims to gather the facts about damage and self‐healing, SH, in Pb‐based halide perovskites (HaPs). Then we seek through reasoning to deduce the implications of these facts. Ultimately, we aim to describe the nature of the damage and SH in HaPs, addressing what appears to be the primary challenge for using HaPs in light ←→ electrical energy conversion and radiation/particle detection, viz., stability. Given the complexity of the topic, a broad range of information must be considered simultaneously. We therefore divide this work into two parts. The first one, presented here, establishes a framework linking macroscopic observations to microscopic mechanisms of damage and healing. The second one will use this framework to develop a model describing how HaP properties evolve under illumination and during healing. In Figure 1 we show a simple scheme that may help the reader to navigate this article by listing most of the important elements that will be discussed.

As far as we know, the possibility of SH in HaPs was first mentioned in 2015 [1] and deduced from experimental results on HaP PV cells in the same year [2]. The following year saw a number of papers showing SH behaviour in PV cells [3, 4, 5], including one showing recovery after exposure to high‐energy radiation (proton [6] beams).

**FIGURE 2:**
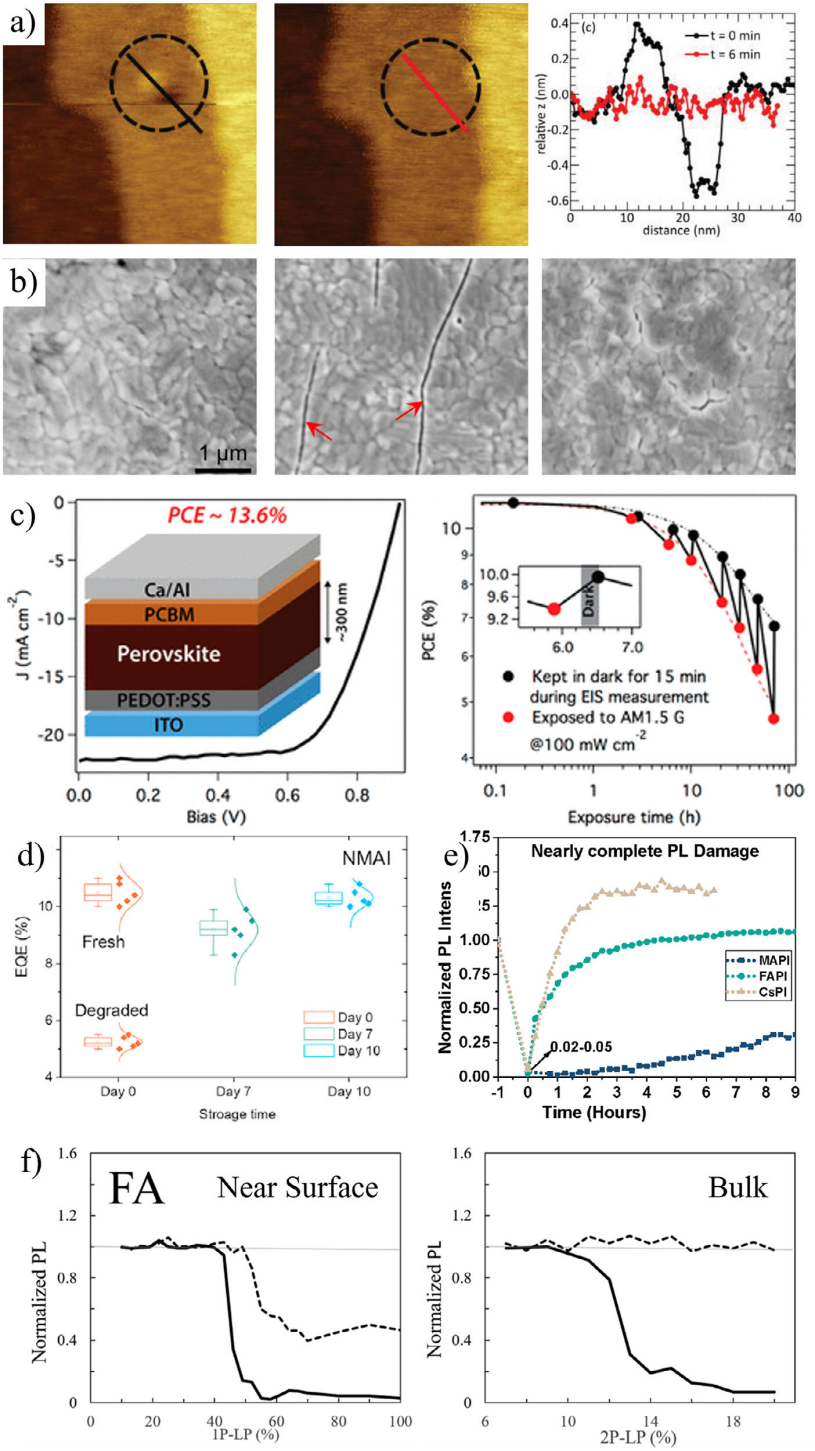
(a) AFM morphology images [17] of a CsPbBr_3_ single crystal immediately after indentation (left) and after a 6 min interval (center). The red and black line profiles (right) show that the original indentation fades within six minutes, restoring a flat surface. (b) SEM images [18] of different MAPbI_3_ perovskite film areas, cracked by tensile stress: before (left), immediately after stress (center), and after 5 min at 100°C (right). The cracks merge over time, effectively healing the broken bonds. (c) Power conversion efficiency (PCE) evolution of a MAPbI_3_‐based solar cell (structure shown at left) under illumination (red dots) and immediately after 15 min in the dark (black dots) [2]. The alternating light–dark periods demonstrate multiple rounds of partial SH, with cumulative degradation. (d) External quantum efficiency (EQE) versus time statistics [19] from five LEDs with a 2D ‐ 3D NMA_2_FA_n_PbI_3n+1_ HaP (with FA as 3D cation and 1‐naphthylmethylammonium iodide (NMAI) as organic spacer 2D cation), showing an initial drop in PCE followed by partial recovery within 7 days and complete recovery in 10 days. (e) Time‐dependent, normalized *PL* measured after light‐induced damage at *t* = 0 in polycrystalline thin films of MAPbI_3_, FAPbI_3_, and CsPbI_3_ [20]. The *PL* signal gradually increases after the damage, with the specific HaP composition strongly influencing recovery kinetics. (f) Power‐dependent photodamage (thick line) observed in single crystal FAPbBr_3_ in the near surface region (left) versus that in the bulk (right) [21]. Near the surface, a clear threshold leads to irreversible damage upon exceeding a critical intensity, causing decomposition. No such threshold appears in the bulk, as there is no material loss to the environment. After 12 h, the *PL* recovers to the undamaged state (indicated by the dotted line). Images adapted from references [17, 18, 19, 20, 21].

SH follows several types of damage, including mechanical (Figure 2a,b), device operational (PV, Figure 2c; LED, Figure 2d), and strong illumination‐induced (Figure 2e, showing SH kinetics, and Figure 2f, showing differences in SH between near‐surface and only bulk absorption in single crystals).

This article is not a comprehensive review of publications on HaP SH, because several such “regular” reviews appeared over the past half‐decade [7, 8, 9, 10, 11]. Instead, we focus on the fundamental, mainly chemical mechanisms underlying intrinsic SH, involving the re‐formation of broken chemical bonds. We exclude SH in organic materials [12, 13], and do not discuss “SH” promoted by polymer additives [14, 15] which should be defined as self‐repair [16].

Therefore, we concentrate on reports relevant to the chemistry of the autonomous recovery of damage of a compound by itself, that is, SH. Our emphasis is on the materials per se rather than on devices, except where relevant to the materials. We begin by presenting established facts, summarizing observations made by the scientific community, and distinguishing verified results from interpretations.

After outlining the thermodynamic background and definitions, we examine the types of damage that APbX_3_ materials can suffer, linking macroscopic observations to demonstrated or likely microscopic and atomistic explanations. We then describe partial or complete recovery during or after damage from both macroscopic and microscopic perspectives. Finally, we explore the implications of these atomistic interpretations, reconnecting them with the facts discussed earlier to derive key insights.

In the last section and the conclusion, we address important issues, such as differences between defect tolerance, self‐repair, and SH [16], and show how faster SH correlates with improved optoelectronic performance.

## 2 Facts/Observations

Our selected key observations on damage and SH in HaPs are presented below, with bold headings to define terms and avoid misinterpretation. This approach follows a recent editorial and earlier work cited therein [22, 23]. By maintaining this separation, we aim to rationalize the observed phenomena so as to enable those that read this review to explain most reported facts at least qualitatively. The forthcoming second modeling article will provide quantitative insights and guidance for future experimental studies.

### 2.1 Accumulation

When HaPs are exposed to external stimuli, they do not change their state instantaneously but instead accumulate “damage”. This is reflected in phenomena such as a decrease of light‐to‐electrical power conversion efficiency (*η*) and reduced *PL* intensity as reported in all the works cited in this section hereafter.

### 2.2 Limitation

In most cases, damage does not progress indefinitely but stabilizes at a functional, though degraded, state (*PL* or *η*) [3, 4, 5, 24, 25, 26, 27, 28, 29, 30, 31, 32, 33, 34, 35, 36, 37, 38, 39, 40].

### 2.3 Enhancement

In certain samples, performance improves if exposed to the same illumination that degrades other samples, a phenomenon that is often called “light‐soaking” [5, 19, 20, 21, 28, 30, 31, 33, 35, 36, 37, 38, 39, 41, 42, 43, 44, 45, 46, 47, 48, 49, 50, 51].

### 2.4 Return

Many devices partially or fully recover their original performance after the cause of the damage is removed [3, 4, 5, 19, 20, 21, 24, 25, 26, 27, 29, 33, 34, 38, 39, 40, 41, 42, 43, 52, 53, 54].

### 2.5 Complexity

Some samples exhibit highly complex behavior during damage, with *PL* or *η *fluctuating, first increasing, then decreasing, or vice versa [3, 19, 20, 21, 41, 42, 43, 47, 51, 54].

### 2.6 Time Scales

The typical time scales for damage and/or SH range from seconds [25, 30, 31, 32, 34, 35, 49, 50, 55], to minutes [3, 4, 20, 21, 24, 37, 38, 41, 42, 43, 44, 46, 47, 48, 53], hours [19, 20, 21, 27, 33, 39, 40, 41, 45, 46, 56, 57], or days [5, 27, 29, 40, 58].

### 2.7 Inter‐Sample (Ir)Reproducibility

Samples processed under similar conditions often show different behavior, even under comparable stimuli [59]. Reproducibility can be improved by comparing results from different groups using nominally identical materials. While not always evident in papers, the issue is recognized in the field and highlighted in a recent primer [60]. (Better still are cross‐lab comparisons of given samples).

### 2.8 Environmental Irreproducibility

Variations in exposure to (different) ambients during testing can affect sample behavior significantly. Studies examining differences in degradation and their evolution confirm this phenomenon [25, 26, 37, 45, 46, 52, 58, 61, 62].

### 2.9 Chemistry Relevance

Despite general irreproducibility among samples, certain compositions perform and recover from damage [21, 28, 39, 42, 54] better than others, consistent with specific chemical species enhancing/impairing HaP performance.

### 2.10 Confinement

A key requirement for SH to occur is that the HaP material, especially in polycrystalline form, is not exposed to the ambient, to prevent any loss of material or reaction with external species [41]. Once material is lost, the material can no longer return to its original chemical state, which often prevents recovery of the relevant function. To that end, samples should be encapsulated, or methods used that only probe their interior [63].

## 3 Consequences

These observations lead to fact‐based conclusions. For completeness, we also note improbable explanations that could negate these conclusions, to counter confirmation bias as part of our approach.

### 3.1 Multiple Processes

External stimulation can lead to at least two opposing processes in HaPs: one decreases and the other increases (PL or *η*) efficiency, probably corresponding to creation/activation and passivation/annihilation of optoelectronically active defects. There may be additional processes and defect types affecting performance.

### 3.2 Chemical Origin

The time and length scales, and chemical sensitivity of *PL* or *η* variations, support a chemical origin involving nuclear motion and bond breaking/forming. Physical processes such as energy or electron transport may occur, too, but they are less environment‐dependent and are orders of magnitude faster.

### 3.3 Steady‐State

Damage is cumulative, and when defect creation and healing rates balance, a new steady state is reached. This state depends on initial conditions and environment, may take hours to form, and can evolve as conditions change.

### 3.4 Non‐Linearity

Small variations in preparation, treatment, or ambient conditions can cause large changes in HaP behavior, indicating strong, non‐linear, sensitivity to chemistry. Resulting *PL* or *η* variations are comparable in magnitude to those defining baseline performance.

## 4 Further Background

Having established the facts and their consequences, we provide additional background by defining relevant terms and the scope of this review. We highlight key studies reporting dynamic perovskite behavior under various stresses and their return to the status quo ante.

We identify damage‐causing factors and analyze their microscopic origins, linking them to macroscopic phenomena, rationalizing each step. We note that while some aspects of HaP behavior are widely accepted, others rely on models that still require experimental validation, which calls for mechanisms for the models to validate or challenge them. Experiments of this type remain rare.

Notably, HaP behavior under damaging conditions is often attributed to defect tolerance (DT) rather than SH [16, 64]. The critical distinction between DT and SH, and why DT alone is insufficient, has been discussed elsewhere [16]. Although experimental evidence for SH is substantial, its theoretical foundation remains limited, whereas the opposite is true for DT.

## 5 Defining Key Concepts

Self‐repair requires additional material and/or energy and external intervention to restore a damaged system.

SH means that recovery is fully autonomous: no external material or factor is needed, and the system returns to its stable thermodynamic (TD) state after damage pushed it out of that state. This implies TD control on time scales relevant for the function of interest of the material rather than geological time scales.

To be relevant, TD equilibrium must be reached rapidly, within hours for PV or milliseconds for detectors, requiring activation energies of only a few kT [65]. For HaPs that self‐heal near room temperature, this corresponds to a few tens of meV.

HaPs are “soft” materials, meaning that they have low elastic constants and hardness. Such properties are critical as they imply the above‐noted small activation energies at ambient temperatures; they derive from the strong lattice dynamics, including “breathing” of the octahedra themselves and one with respect to the other, as well as A‐cation motion. All these properties lead to a shallow energy landscape (but loss of material or reactions with external species changes the local landscape).

Thus, HaPs are TD‐controlled materials that dynamically respond to external conditions. We distinguish between dynamic processes that enhance or degrade optoelectronic performance, with *PL* changes serving as indicators. Practices such as light soaking can shift HaPs into more favorable states, reversibly or irreversibly.

### 5.1 Phenomenology of Self‐Healing of HaP Materials

This section presents key experimental data on SH. In our work, damage was induced by intense light; other causes include high‐energy radiation or particle beams (X‐, *γ*‐rays, protons, positrons, electrons), mechanical stress, and electric fields.

Mechanical damage is the simplest case mechanistically. Healing of cracks or morphological changes requires some migration ability of all constituents. In an AFM study where damage was induced by the tip, SH occurred within minutes to tens of minutes, with ∼0.5 nm topography changes, consistent with diffusion of the slowest species, likely Pb^2^^+^ (BOX 1) [17].

From our studies of SH kinetics in various single‐phase HaPs, the healing time increases with damage severity, consistent with greater damage accumulation [20, 41, 57]. For weaker damage, such as in PV cells under normal illumination, SH may be efficient enough to counteract damage as it occurs, maintaining a (near)equilibrium‐induced defect density. In an early study, Nie et al. [3] showed that MAPbI_3_ PV cells exhibited an ∼8% photocurrent drop under sunlight, which the authors attributed to light‐induced traps, followed by recovery in the dark. SH occurred in two steps: ∼30 s for two‐thirds of the loss and ∼1 h for the remainder, with strong temperature dependence.

Based on SH time scales in these experiments (typically hours for moderate to severe damage), we argue that diffusion of certain species is the rate‐determining step. For 2D/3D HaPs, we showed that low damage (∼25% *PL* loss) can fully heal within 10–100 s [43], implying that performance‐limiting defects form and disappear rapidly and therefore involve fast‐diffusing species. This points to iodine and proton species as the likely damage agents (BOX 1), often described as vacancies or interstitials. At a first level of analysis, simple SH mechanisms may include iodide migration to fill iodide vacancies or reaction with Pb^0^ formed under illumination, analogous to PbI_2_ photolysis (PbI_2 _+ 2hν ↔ Pb^0^ + I_2_). Given the low likelihood of stable isolated point defects in the dynamic HaP lattice, complex mechanisms are probable, with, for example, polyhalide anions (see section 5.4). Notably, SH kinetics depend strongly on HaP composition, particularly the A cation. For example, FAPbBr_3_ crystals heal much faster (seconds) than CsPbBr_3_ ones (hours) [21, 41]. In MAPbI_3_, the presence of water both reduces damage and accelerates SH [42], and simulations show that water lowers the activation energy for iodine diffusion [66].

A second hypothesis from our studies of strong light‐induced damage links lattice strain to the SH rate. FA‐based HaPs, or MAPbI_3_ with 15%–20% MA replaced by larger cations (guanidinium or acetamidinium), heal faster than pure MAPbI_3_ [57]. These larger cations induce tensile strain in the lattice. Although the mechanism is not fully understood, tensile strain may lower activation energies for bond‐breaking and re‐formation, as suggested by studies where increased tensile stress accelerated light‐induced HaP decomposition [67]. Because SH involves bond re‐formation and ion motion, strain‐induced vacancy formation and diffusion may also accelerate SH. These interpretations remain partial and require further experimental validation.

SH under high‐energy radiation remains intriguing and underexplored. The first report involved 68 MeV proton irradiation, yielding a rapid (∼3%) current recovery within ∼12 min after irradiation ceased, attributed to quickly repairable defects [6]. However, SH often continues for days after high‐energy exposure, indicating multiple recovery timescales [68, 69, 70].

Interestingly, higher‐energy radiation can induce SH. Soft X‐rays damage FAPbBr_3_ by forming Pb^0^ and causing Br_2_ loss, whereas intense X‐rays stimulate a process that re‐oxidizes Pb^0^ consuming H^+^ and restores FAPbBr_3_ (even if it cannot proceed indefinitely) [44]. Analogously, low‐energy protons damage HaP solar cells, while higher‐energy protons generate local heating that accelerates SH [68]. FAPbI_3_ stands out for not forming Pb^0^ under intense γ‐ray doses, implying an especially robust SH mechanism [71].

Pb^0^ re‐oxidation, likely via reactions with oxidized halide species, is central to many SH processes. Overall radiation resilience may arise from radiation hardness (strong chemical bonds), intrinsic defect tolerance (mainly very shallow defect energy levels), or rapid SH to maintain low defect densities; the latter is most plausible and implies both fast and slow SH components.

**BOX 1: Ionic diffusion coefficients in Pb halide perovskites**

What moves in Pb‐halide perovskites, besides energy and electronic carriers? Given the partially ionic nature of these materials, this question can be reframed as: Which ions are mobile? Given their partially ionic nature, this question concerns ionic mobility on time scales relevant to SH. We focus on hybrid organic–inorganic perovskites, particularly MAPbI_3_. Protons are prime mobile candidates, originating from MA^+^/FA^+^ deprotonation or traces of water [72, 73, 74, 75, 76]. Early estimates discounted proton diffusion based on MA^+^ acidity in water, but later work suggests higher proton concentrations [72, 73, 74, 75, 76]. Halide ions, especially iodide, are also likely mobile species.

Several years ago, we reviewed relevant experimental results (including our own) on ion diffusion in HaPs [73]. We noted that many reports of high ionic diffusivity (with diffusion coefficients ranging from ∼10^−7^ to 10^−10^ cm^2^ s^−1^), originally attributed to iodide migration, likely reflect instead proton transport. This mis‐attribution arose because common electrical characterization techniques, such as impedance spectroscopy, are unable to distinguish between different ionic species. Nonetheless, halide migration, even at slower rates, is essential to explain phenomena like halide segregation observed in mixed iodide–bromide compositions. In these cases, diffusion coefficients in the range of ∼10^−11^ to 10^−14^ cm^2^ s^−1^ have been deduced [73]. From classical impedance studies [77] on PbI_2_ an iodide self‐diffusion coefficient of 10^−18 ^cm^2^s^−1^ at RT can be extrapolated from data at 245°C–300°C. If the presence or formation of the trihalide (tri‐iodide) species is considered, iodide migration can be more efficient than found from these types of measurements, with a Grotthuss‐like, caterpillar mechanism.

Other candidate ions include MA^+^ and Pb^2^^+^. MA^+^ diffusion was directly measured [78, 79] by nuclear magnetic resonance (NMR) with ^1^ ^3^C‐ and ^1^ ^5^N‐labeled MA^+^, establishing an upper limit of ∼10^−15^ cm^2^ s^−^^1^. Interdiffusion experiments tracked by time‐of‐flight secondary ion mass spectrometry (ToF‐SIMS) gave an MA^+^ bulk diffusion coefficient of ∼3 × 10^−17 ^cm^2^ s^−^^1^, with faster diffusion (>10^−13^ cm^2^ s^−^^1^), assumed along grain boundaries [79]. Other studies, using capacitance‐based transient ion drift [80] or deep‐level transient spectroscopy [81], also discuss MA^+^ diffusion. They observed a fast (with diffusion coefficients of 10^−8^ to 10^−9^ cm^2 ^s^−1^) and a slower ionic component (with diffusion coefficients of 10^−11^ to 10^−12^ cm^2 ^s^−1^), attributing the faster diffusion to iodide and the slower one to MA^+^. We posit that the faster component should instead be assigned to proton diffusion, the slower one to iodide migration, and that MA^+^ (and Pb^2+^) diffusion will be even slower.

Direct evidence for MA^+^ diffusion exists only from labeled MAPbI_3_ studies [78, 79]. Pb^2^^+^ diffusion is proposed at times, based on theory or indirect arguments, including NMR‐based ones, such as those discussed in ref [82]. However, radioactive tracer experiments in Pb dihalides strongly argue against significant Pb^2^^+^ mobility (note: PbI_2_ has a CdI_2_ structure, related to the perovskite one (edge‐sharing metal halide octahedra), but the PbBr_2_ structure does not resemble the perovskite one). Early ^2^ ^1^ ^0^Pb studies in PbI_2_ extrapolate to D(Pb^2^^+^) ≲ 10^−^^1^ ^8^ cm^2^ s^−^^1^ at 25°C–40°C [83, 84], while later tracer data in PbBr_2_ give D(Pb^2^^+^) ∼10^−^^1^ ^5^ cm^2^ s^−^^1^ and D(Br^−^) ∼10^−^^1^ ^3^ cm^2^ s^−^^1^ at 40°C [85].

This short survey suggests that Pb‐diffusion in the Pb‐HaPs is unlikely to be important, at least in the bulk of the material/grains. However, as the experiments of fracture healing show, some movement must occur to allow re‐bonding of broken parts. Such a process may occur at the surfaces, where mobility can be higher than in the bulk, as shown by the above‐noted ∼10^4^× higher grain than bulk MA+ value.

To understand SH, we need to know the nature of the damage. To that end, we will now discuss what is known or conjectured to cause damage to HaPs and what is happening macroscopically under different stimuli. We then turn to the main topic: SH in HaPs, also adding a discussion of photo‐brightening (increase in *PL*).

### 5.2 Macroscopic View

Because HaPs are mainly studied for light ←→ electricity conversion and for radiation/particle beam detection, radiation and thermal damage are the most investigated. In some cases, “damage” may be a misnomer, as certain treatments improve performance. Here, we use “damage” broadly to denote any deviation from the initial stable thermodynamic state. Damage can be classified as reversible or irreversible: reversible damage can self‐heal when external conditions are restored, whereas irreversible damage cannot.

#### 5.2.1 Mechanical Damage

Mechanical damage is the simplest case and shows surprising reversibility, particularly relevant for macroelectronics such as flexible solar panels. Stress‐induced cracking or scratching [25] can partially heal via SH [17, 18, 86]. This process resembles room‐temperature annealing or diffusion bonding in metals: HaPs can re‐form bonds, and even cleaved single crystals can re‐bond when pressed together.

#### 5.2.2 Temperature‐Induced Damage

**FIGURE 3:**
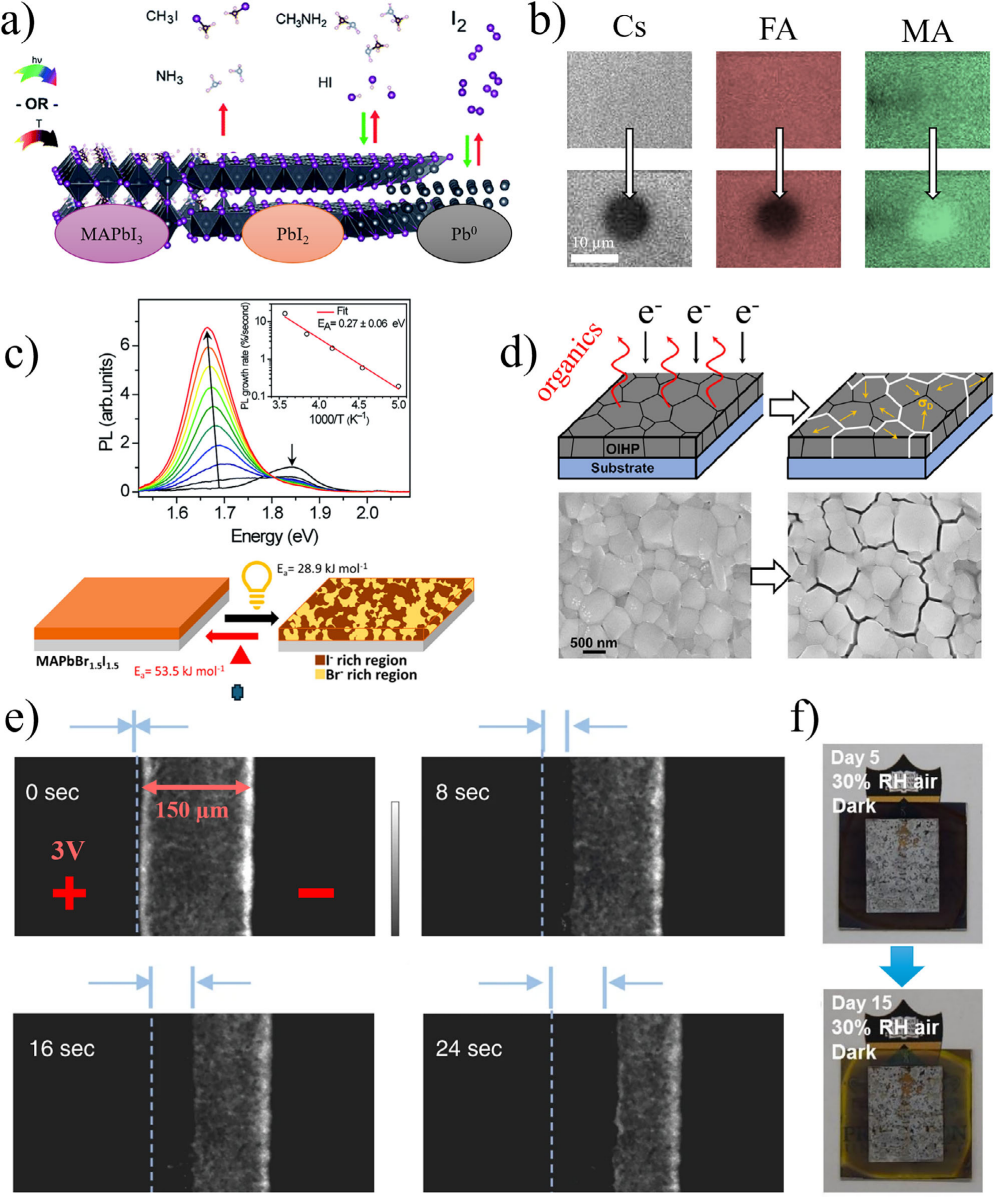
(a) Stages of thermal and light‐induced decomposition in MAPbI_3_, which leads to PbI_2_ and Pb^0^ as remaining solid phases (right) if the perovskite irreversibly loses ammonia and iodomethane (left), while reversibly desorbing methylamine, hydroiodic acid, and iodine. (b) 2‐Photon light‐induced damage inside three crystals of APbBr_3_ HaPs with different A^+^: Intense local illumination modifies the original HaP *PL* (top row), indicating localized damage (bottom row). (c) Mixed‐halide perovskite demixing under illumination: The perovskite separates into iodine‐rich and bromine‐rich phases, with the *PL* spectrum red‐shifting as the iodine‐rich phase emits most of the light. (d) Vacuum and electron‐beam damage in electron microscopy: Material loss and subsequent cracking occur due to induced stress, resulting in irreversible damage. (e) Electric damage: on a MAPbI_3_ thin film are deposited 2 Au electrodes at a 150 µm distance, and 3 V are imposed between them on one side (left), which leads to defect formation and decreased *PL*. The front of the darkening advances toward the right over time. (f) Damage in MAPbI_3_ devices due to humidity and Al contacts: The result is a corrosion‐like mechanism, damaging the layer by aluminum ions and water. Images adapted from references [42, 87, 102, 105, 116, 127, 130].

Temperature‐induced damage is the simplest type to investigate (Figure 3a) [87]. It results from shifts in thermodynamic equilibrium and increased vapor pressures of species such as bromine, iodine, methylamine, and iodomethane at elevated temperatures [88]. In confined environments, released species can be reabsorbed, restoring equilibrium. However, at sufficiently high temperatures, irreversible chemical reactions may occur [88], particularly above ∼200°C (notably for formamidinium) [30, 89, 90] though these are less relevant under typical operating conditions for HaP devices.

#### 5.2.3 Light‐Induced Damage

Beyond thermal effects, light‐induced damage is widely studied in perovskites (Figure 3b) [27]. Illumination often changes *PL* and light‐to‐electrical power conversion efficiency (*η*), reflecting shifts in the perovskite state [46]. If no material is lost, self‐healing is in principle possible. However, preventing material loss is difficult because perovskites can decompose into halogens that react with metals, dissolve in plastics, and interact with organic materials. Since encapsulants, electron transport layers (ETLs) and hole transport layers (HTLs) often contain polymers or conjugated materials, irreversible halogen loss under illumination can cause permanent damage.

Engineering solutions, such as avoiding direct contact with metals and encapsulants, selecting ETLs and HTLs that do not react with halides, or introducing catalysts to reconvert halogens into halides, make it possible to mitigate this, rendering light‐induced damage potentially reversible. Light‐induced damage may also include thermal contributions or arise from direct photochemical processes, especially under UV irradiation.

Under tight, non‐reactive encapsulation, decomposition products may recombine to re‐form the original HaP, although internal morphology will change due to recrystallization. We demonstrated this by converting a HaP single crystal into a plasma using an intense pulsed laser, which decomposed and re‐formed as small HaP crystallites filling the original volume [41].

Without effective encapsulation, that is, if material can escape or react with contacting media, photolysis, similar to the known PbX_2_ → Pb^0^↓ + X_2_↑ light‐induced decomposition, is driven forward. While isolated Pb^0^ atoms are unstable (except in organometallic complexes) [91], nanodomains containing tens of Pb atoms may form (the smallest isolated Pb nanoparticles reported, contain several 1000 s of atoms) [92], though this would require extreme conditions given limited Pb diffusion. More plausibly, halogen loss (e.g., I_2_) leaves excess electrons, n‐doping the material or reducing p‐doping. Since bona fide bulk‐doped p–n junctions have not been realized in HaP devices (where doping seems to be actually dominated by surface defects) [93], such doping might exceed the HaP's stability range. These considerations are important for interpreting photolysis with halogen loss from microcrystals or polycrystalline films under illumination (e.g., refs. [94, 95]).

#### 5.2.4 High‐Energy Radiation Damage

When HaPs are exposed to X‐rays [96, 97] or γ‐rays [98, 99], they suffer damage qualitatively similar to that induced by light. Such exposure can lead to Pb^0^ formation, material loss, and, in some cases, phase‐separation. In some conditions, however, they can induce the irreversible radiolysis of the organic cations [96] or structural modifications [99].

#### 5.2.5 Vacuum‐Induced Damage

For electron microscopy [100, 101, 102] and other techniques requiring high or ultra‐high vacuum (e.g., photoemission and ToF‐SIMS), it is important to distinguish particle from vacuum‐induced damage. The latter is irreversible, as volatile components such as halogens or methylamine escape under vacuum and are removed from the HaP (Figure 3d) [102]. Irreversible loss of material causes changes that cannot be reversed autonomously.

#### 5.2.6 Particle (Electrons, Positrons, etc.) Damage

Electron beams in SEMs or TEMs can damage HaPs by depositing energy and locally altering charge distributions. This damage may be reversible, depending on beam intensity and exposure. Positively charged particles (protons, positrons, ion beams) also induce damage, with effects differing from electrons. Low‐energy proton beams (tens of keV) can cause ion displacement, whereas higher‐energy beams (≥1 MeV) deposit energy mainly as heat, generate fewer vacancies, and can even accelerate SH [68].

#### 5.2.7 Electrical Damage

In applications such as LEDs or ion‐migration studies, high applied voltages can induce electrical damage, which can be divided into two types. The first arises from the migration and accumulation of ions near the electrodes. Although this can modify *PL* (Figure 3e) and device efficiency, it is in principle reversible, since ions can diffuse back to their original positions once the field is removed. Accordingly, voltage‐induced changes during ion‐migration studies often partially reverse [40] or fully recover after bias removal [19], similar to electrochromic windows and light‐emitting electrochemical cells (LECs) [103, 104]. In contrast, electrical damage becomes irreversible when electrochemical reactions resembling electrolysis occur, leading to the formation of metallic Pb and halogen gases [19, 105, 106, 107, 108, 109, 110]. Because hybrid perovskites allow a wider range of electrochemical reactions, fully inorganic compositions are expected to be more stable under such stress. Under controlled conditions, however, this damage may still be partially reversible.

Importantly, closely related degradation processes can also occur in perovskite solar cells subjected to reverse‐bias conditions, for example, during partial shading of a module [111]. In this case, reverse bias leads to strong local electric‐field amplification due to ionic accumulation, particularly at interfaces and grain boundaries [112]. This electric field may promote localized electrochemical decomposition, electrode redox reactions, and the formation of conductive shunt pathways. Although engineering solutions exist to mitigate reverse‐bias stress [113], the underlying damage mechanisms are not yet fully resolved experimentally. Evidence indicates that degradation is highly localized, predominantly at grain boundaries and contact interfaces, and is chemically consistent with ion‐driven electrochemical breakdown processes similar to those observed in electrically stressed perovskite LEDs. This damage favors the reactions illustrated in the redox process paragraph of the microscopic view of damage section.

#### 5.2.8 Chemical Damage

Chemical interactions can also cause damage. While HaP modification due to exposure to O_2_ or humidity below ∼40% RH is often reversible, metal electrodes can diffuse into the HaP, likely via grain surfaces and boundaries, degrading functionality. Some HTL [114] or ETL materials in direct contact with the HaP may react with it. Such processes, that can be viewed as corrosion at material interfaces [115, 116, 117], are generally irreversible and can lead to permanent compositional changes (Figure 3f). Although sometimes called chemical “doping,” “alloying” is a better term because of the high concentrations involved. The role of (photo)(electro)chemical processes in HaPs and devices was discussed in a recent perspective [118].

#### 5.2.9 Phase Change and Phase Separation

Phase change is another form of damage. For example, water exposure can rapidly convert α‐FAPbI_3_ or γ‐CsPbI_3_ from black to yellow δ phases [119, 120]. These transitions are irreversible at room temperature, as the system moves to a more stable thermodynamic state, even though additives can sometimes stabilize the α phase or enable limited recovery [121]. We do not focus on this damage or SH here and mention it only for completeness.

Another form of light‐induced damage is phase separation (demixing) in mixed‐halide, esp. (Br,I) HaPs (Figure 3c), where illumination drives the formation of nano‐ to microscale regions with different halide compositions [122]. This process is driven by photo‐generated charge carriers, which, in (Br,I) compounds lower the free energy of iodide‐rich regions, making them energetically favorable under illumination. As a result, halide ions migrate to form I‐rich and Br‐rich domains [123, 124]. This alters the optoelectronic properties from those of the original homogeneous material and typically reduces device performance, as the initial composition was optimized. Importantly, this damage is often reversible; that is, after removing illumination, the HaP can return to a fully mixed state within minutes [125, 126].

Halide segregation under illumination was first demonstrated in 2015 [127], when it was shown that MAPb(I_1__−__x_Br_x_)_3_ segregates under light and remixes in the dark within ∼5 min. While the light‐induced segregation mechanism has received extensive attention, the dark remixing process, which we regard as a form of SH, has received far less attention. The prevailing view is that remixing is driven primarily by entropy gain, leading to a lower free energy of the mixed phase, possibly assisted by lattice strain. Thermodynamic calculations [123, 128] predict stability of mixed MAPb(I_1__−__x_Br_x_)_3_ up to *x* ≈ 0.2–0.33, while experiments show segregation above *x* ≈ 0.2 [127], consistent with these predictions. Importantly, halide segregation is not restricted to Br‐rich compositions under illumination; electrical bias can also induce segregation even in mixed‐halide perovskites with low Br content (*x* ≈ 0.1) that are otherwise optically stable. In such cases, the onset of bias‐induced segregation has been linked to iodide/triiodide/iodine electrochemistry and coupled redox/doping reactions in device stacks, highlighting that “segregation” under operating conditions can be intertwined with interfacial redox processes rather than being purely a photo‐thermodynamic effect [129].

The activation energy for segregation in MA‐based mixed HaPs was found to be lower than that for remixing [130, 131], while in another study [132] (using a triple cation HaP instead of the single MA cation), the opposite was found, although the segregation process was still much faster than the remixing. Elmelund et al. linked the rate of segregation to the rate of halide diffusion which has been reported to be increased by light [133, 134]. Structural factors may also play a role: iodide‐rich regions are expected to adopt a tetragonal phase with higher halide mobility than the more bromide‐rich cubic regions, which may influence both segregation and remixing kinetics [135].

Chemical evolution can sometimes be mistaken for self‐healing. For example, in phase‐separated samples, loss of I_2_ in non‐encapsulated samples from iodide‐rich regions can lead to an (indirect) reduction of the‐low energy *PL* peak. This low‐energy peak is a typical marker of segregation, and its reduction could be mistaken for SH [94]. Such effects can be significant in phase‐separated systems.

From our perspective, the key point is that dark remixing following light‐induced segregation constitutes a clear example of intrinsic SH, as the system autonomously returns to its original chemical state once the perturbation is removed.

### 5.3 Microscopic View of Damage

Macroscopic damage and SH in halide perovskites must reflect changes at the atomic level, including bond breaking and formation, as well as ionic or molecular rearrangements. Although these processes have major electrical consequences, the damage is intrinsically chemical and cannot be understood without considering the underlying chemistry. Because Kröger–Vink notation for static point defects is often unfamiliar and prone to over‐interpretation, we do not use it here. Instead, Box 2 provides background linking this notation to the perovskite chemical environment, aiding comparison with DFT studies, which commonly employ it. In doing so, the highly dynamic nature of the halide perovskite lattice, which complicates the concept of static defects [65, 136], should always be kept in mind.

Damage at the atomic level typically involves bond breaking, which can either be an electrochemical, redox process, if it involves Pb─X bonds, or, for hybrid HaPs, a non‐electrochemical, for example, acid‐base process related to the proton chemistries of methylammonium, formamidinium, or other ammonium ions in the lattice.

We describe atomic‐scale processes using two complementary chemical models: one focused on bond breaking and reforming, and another on the chemical species that emerge as a result of these processes. Broken bonds correspond to point (0‐D) defects such as vacancies, interstitials, or antisite defects, which fluctuate dynamically and therefore lack well‐defined energy levels [137, 138, 139]. Here we focus on MAPbI_3_ as a prototypical system, noting that the same principles extend to other halide, cation, and mixed‐composition perovskites, with the exception that Cs‐based materials lack proton chemistry, (apart from that due to H_2_O traces). The chemical properties and defect behavior of mixed‐cation and mixed‐anion perovskites are expected to be similar to those of the simpler HaPs.

### 5.4 Redox Process

**FIGURE 4:**
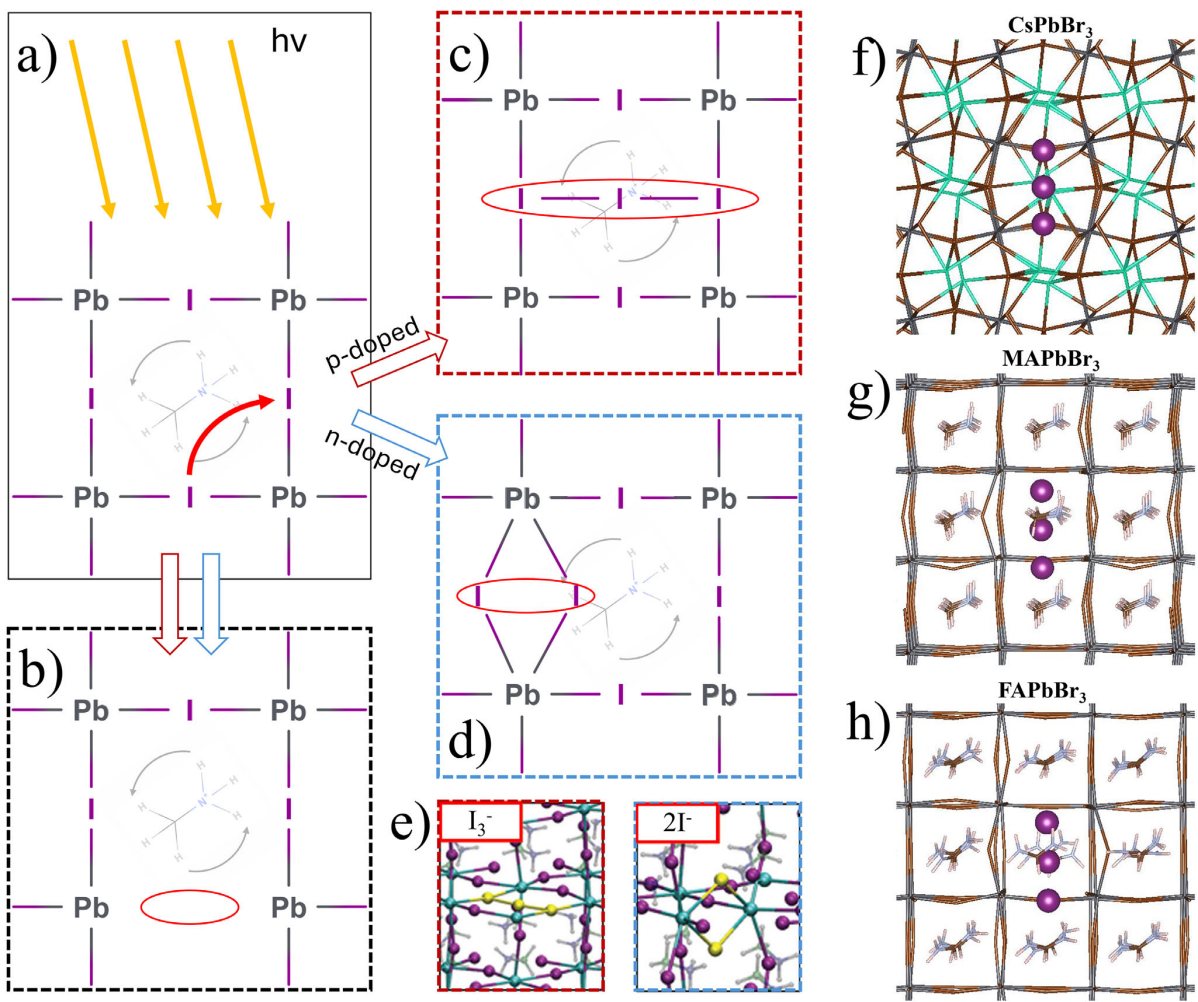
Two‐dimensional projected visualizations of light‐induced damage and subsequent self‐healing in Pb‐halide perovskites. (a) Light is absorbed by the perovskite which causes the movement of the halides with one binding to another. (b) One halide leaves a vacancy behind. (c,d) Show the left side of (a) and the next I–Pb section of the lattice. (c) If the perovskite is (weakly) p‐doped (as is mostly the case) an $X_{3}^{-}$ species forms, corresponding to the species in solution where two formally neutral I connect to an iodide ion. (d) If the perovskite is n‐doped a different interstitial defect forms. In (a–d) the methylammonium ion is semi‐transparent to indicate that it is located behind the plane of the atoms considered in the scheme. The structure of these interstitial defects has been calculated by *static* DFT for (e) MAPbI_3_ [140]. (f) CsPbBr_3_, (g) MAPbBr_3_, (h) FAPbBr_3_ [21]. The lack of deformation of the Pb–Br substructure in CsPbBr_3_ indicates the higher stability of the defect compared to MAPbBr_3_ and FAPbBr_3_, where the bromides (indicated as “vertices” of the rods) are not aligned with the ones of the other unit cells (below the one in the plane of the figure). Figures adapted from ref. [140] and ref [21].

Consider breaking a Pb─I bond. The partially reduced Pb remains coordinated to five iodides (PbI_5_), while the partially oxidized iodide becomes under‐coordinated, forming two adjacent distorted PbI_6_ octahedra. These higher‐energy Pb and I species are prone to react or migrate and may separate from the halide perovskite phase. If the bond does not rapidly reform, the under‐coordinated iodide can approach another I^−^ (Figure 4a) and create an iodine vacancy (*V*_i_) (Figure 4b) [30].

The final structure depends on doping [93]. In electron‐poor (p‐doped) lattices, I^−^ typically associates with two iodides (one interstitial) losing 2 electrons to the lattice to form I_3_^−^ (Figure 4c). In electron‐rich (n‐doped) lattices, it instead coordinates with two Pb^2^^+^ ions, displacing a nearby I^−^ via electrostatic repulsion (Figure 4d). As in solution, we do not expect neutral iodine radicals. As Pb─I bonds break, iodide mobility increases, and electrons can move freely through the crystal. DFT structures of these defects are shown in Figure 4e [140]. As their formation energies have only been computed [140, 141] and never measured, the stability ranges of these species remain uncertain.

Chemical intuition suggests that in p‐type lattices, sufficiently large fluctuations allow iodide migration via a Grotthuss‐like mechanism involving I_3_^−^ reorientation. In less p‐doped, or n‐doped lattices, diffusion instead proceeds through electrostatically driven motion of bridging iodides around Pb^2^^+^ ions. Because these mechanisms have different energy barriers, the associated iodide species should exhibit distinct diffusion coefficients.

The choice of monocation in perovskites strongly influences ion diffusion by modifying the lattice structure. DFT calculations of Br_3_^−^ in CsPbBr_3_, MAPbBr_3_, and FAPbBr_3_ (Figure 4f–h) show that the more symmetric and stable CsPbBr_3_ lattice slows ion diffusion, consistent with its slower self‐healing (SH). In contrast, FAPbBr_3_, which shows the fastest SH, has a highly distorted lattice in which Br_3_^−^ is already positioned to migrate into a neighboring unit cell, aided by interaction with a strongly displaced FA cation [21].

**BOX 2: Kröger–Vink notation for point defects in lead‑halide perovskites (APbX_3_)**

In the Kröger–Vink (K‐V) notation the general symbol for a point defect is $A_{S}^{q}$ where *A* is the chemical symbol of the defect species (the element that occupies the site or *V* if it is missing, for Vacancy), subscript *_S_* is the element that would be there in the perfect structure or *_i_* for an interstitial site (a location that would not be occupied in the ideal lattice) and superscript *^q^* gives the charge relative to the formal one for this site in the ideal lattice, where *^q^* can be *^×^*  (= 0), •  (=+1), ′  (=−1), and multiples of • or ′ for multiple charges).

In our discussion, we mention several chemical species that can, in principle, be mapped onto defects using K–V notation, *if we assume a static lattice* at 0 K. As we already emphasized, this assumption does not reflect the physical reality of HaPs, which do not behave as rigid crystals with a static lattice, but as a dynamic, strongly anharmonic one. In such a “liquid‐like” lattice, atoms experience large, thermally driven displacements, and the concept of well‐defined, point‐like defects, if taken literally, becomes problematic. The point is stressed by Poulsen in the introduction to his thesis [142].

A possible analogy is water: in aqueous solution, a proton is better represented as H_3_O^+^ than as a simple interstitial $H_{i}^{•}$. One could argue that in ice at 0 K, $H_{i}^{•}$ might be a meaningful description, but that is not water, but ice, with a fundamentally different structure. Density functional theory (DFT) calculations, which are typically carried out on static supercells at 0 K, face the same conceptual problem. While these calculations provide valuable insight into possible bonding configurations, they cannot account for the strong, complex, simultaneous atomic motions that occur in real water and HaPs.

That said, K–V point defect notation can still be instructive to loosely associate chemical species described in this review with the formal K–V notation used in the DFT literature, if we remain aware that we use a model that assumes the existence of separate, static point defects. This approach can help interpret the results of theoretical studies without mistaking static defect models for physical reality.

With this in mind, we can, for the iodine‐related interstitials and proton‐mediated defects we have discussed, make the following associations:

**Table:** 

*I_i_* *see note* below*	interstitial I; forms a trihalide complex, I_3_^−^, in p‐doped samples; see Figure 4c,e.	$H_{CH_{3}{\mathrm{NH}}_{3}^{+}}^{\;x}$	Proton loosely occupying the former A‐site of a methylammonium (MA^+^); charge‐compensating species; see Figure 5g
$I_{i}^{\prime }$	Additional I^−^ bridging two Pb^2^^+^ ions in n‐doped samples; see Figure 4d,e.	$V_{H^{+}}^{\prime }\left(N\right)$	Missing proton from N–H group of MA^+^; neutral methylamine can possibly interact with a lattice iodide
$H_{i}^{•}$	An additional proton inside the unit cell. See Figure 5d.	$V_{CH_{3}{NH}_{3}^{+}}^{\prime }$	Vacant MA^+^ site at the center of the unit cell
$V_{I}^{•}$	Missing I^−^ originally bridging two Pb^2^^+^ ions; see Figure 4b.	**complex** $\left(V_{H^{+}}^{\prime }V_{I}^{•}\right)$	A defect complex where MA^0^ donates a lone pair to undercoordinated Pb^2^^+^, partially restoring bonding lost because of a $V_{I}^{•};$ chemically this is [MA^0^PbX_3_]^−^; see Figure 5f

^*^In some, especially theory‐related HaP literature, $I_{i}^{•}$, that is, an I^+^ cation, is invoked. We refrain from using this notation as such an ion is not known in chemistry. Even a formal +1 charge for Iodine is extremely rare and then only in highly specialized molecules/ligands. As such use of $I_{i}^{•}$ is a bit like using a fictitious asset in accounting. We use ***I_i_*** instead in this discussion.

**FIGURE 5:**
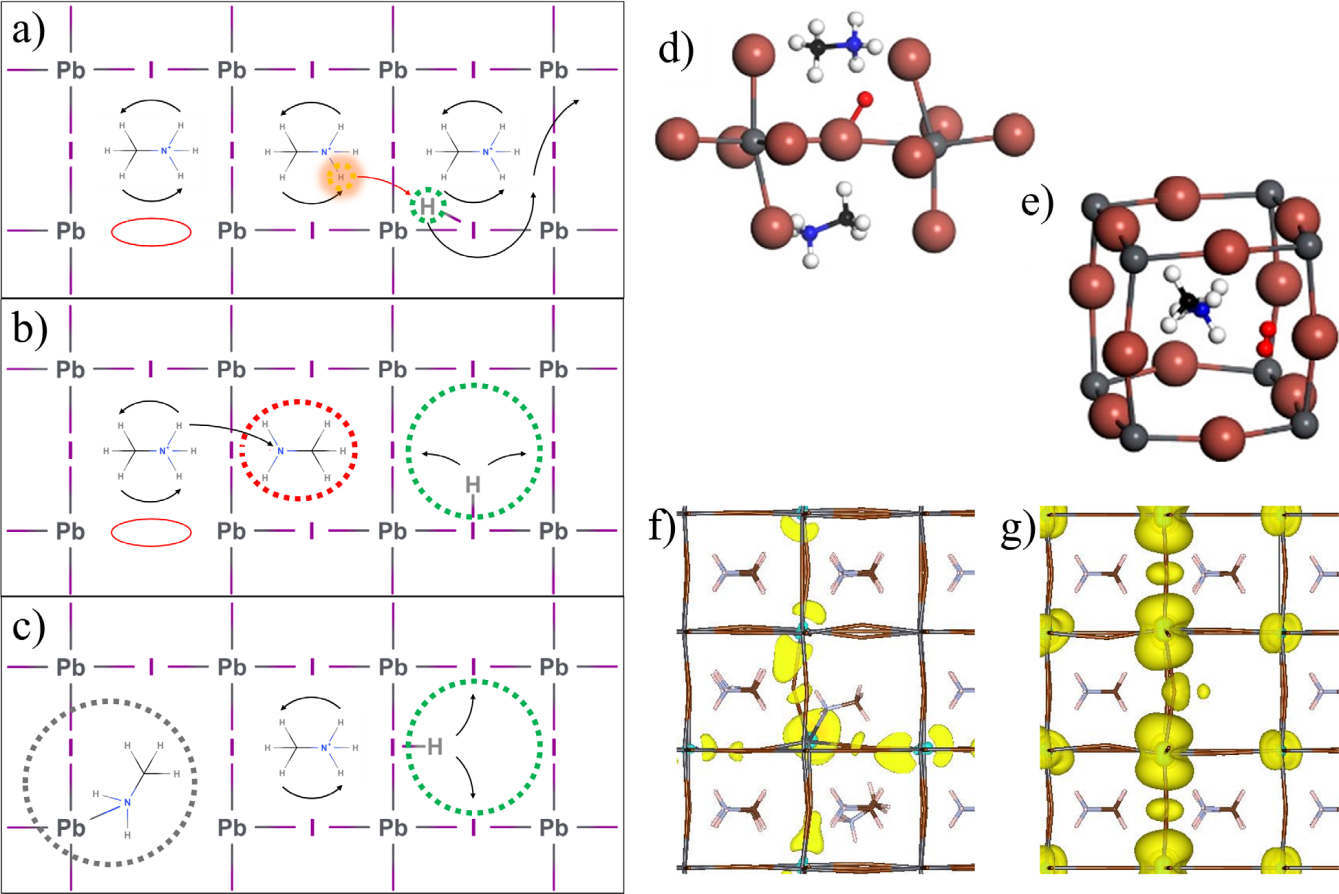
(a) Left: an iodide vacancy (red‐left) is present in a unit cell. Light causes breaking of a methylammonium N─H bond with loss of a proton (orange—center) that becomes an “interstitial” defect in another unit cell (green—right). (b) this proton may encounter/be attracted to a methylammonium vacancy and fill it (green—right). A proton from a methylammonium can migrate to the methylamine that formed upon proton loss (red—center) whose N‐atom is bound to an iodine atom of the structure (bond in blue—center). (c) The methylamine that formed in (b), and coexists in the same unit cell with an iodide vacancy, binds to the lead, passivating that defect (gray‐left) while the proton continues to replace the missing methylammonium (green‐right). (d) structure of the *H^+^* defect (red dot) present for p‐type perovskites. (e) structure of the *H_2_* defect (red dots), present in strongly n‐doped perovskites (if achievable). In (d) and in (e) Pb is in gray and I in red. (f) DFT calculation of the structure of the product of the reaction between methylamine and an iodide vacancy. (g) DFT calculation of the structure of a H^+^ substituting a CH_3_ NH_3_^+^. Figures adapted from refs. [21, 148].

External species such as water, and possibly ammonia or methylamine, can also affect ion diffusion coefficients [66] and defect dynamics. For example, we observe that MAPbI_3_ single crystals show less damage and faster SH at 45% relative humidity than under dry conditions [42].

The behavior of I_3_^−^ species expected under p‐doping differs from that of bridging I^−^ species expected under (speculative) n‐doping. I_3_^−^ can act as an electron trap, capturing two electrons to form a bridging I^−^,

$${\left(I_{3}\right)}^{-}+2e^{-}\to 2I_{I}^{-}+I_{i}^{-}$$

while the reverse process occurs when bridging iodides capture two holes to reform I_3_^−^.

$$2I_{I}^{-}+I_{i}^{-}\to 2e^{-}+{\left(I_{3}\right)}^{-}$$

Whether or not this redox interconversion occurs under illumination, depends on the relative timescales of electronic and ionic processes.

Strong lattice dynamics in halide perovskites complicate this picture, and a unified model coupling charge capture, recombination, and ion migration is still lacking. However, under charge injection (e.g., electrochemical damage), transitions between I_3_^−^ and bridging I^−^ effectively modify doping, a situation further complicated by the presence of additional mobile ions.

Vacancy defects are central to atomistic modelling of SH, as damage‐generated vacancies must recombine with their corresponding species. Halide vacancies, *V*_X_, generally exist in a single charge state and act as electron traps. Although DFT studies often place their energy levels near the conduction band and therefore emphasize interstitial defects, we question this focus and revisit the role of *V*_X_ later in this work.

### 5.5 Bond and Chemical View

Below we provide a concise overview from two complementary perspectives: one focused on bond structure and the other on overall chemistry. At the microscopic level, there is no fundamental distinction between forward and reverse reactions and what we term “damage” and “self‐healing” (SH); SH simply corresponds to a shift of the chemical equilibrium back toward the initial state. Here, X denotes any halide in an APbX_3_ perovskite, and the redox process is outlined schematically.

In the bond‐structure view (Table 1, left), X_6_Pb represents a corner‐sharing (PbX_6_)^2^^−^ octahedron. Electrons released upon Pb─X bond breaking do not initiate radical chemistry, as halide perovskites are semiconductors in which charge redistributes.

**TABLE 1:** Summary of the reactions involved in the formation of halide “vacancy” and “interstitial” defects in HaPs, with the left column illustrating bond cleavage and formation, and the right column presenting the corresponding chemical species.

Bonds	Chemistry
Normal bond: X_5_Pb − X − PbX_5_ Additional X bridging 2 Pb (n‐doping): $X_{5}Pb{<}_{X}^{X}>PbX_{5}$ Additional X bridging 2 X (p‐doping): $\begin{array}{ccc} X_{4}\text{Pb} & -X- & \text{Pb}X_{4}\\ X & -X- & X\\ X_{4}\text{Pb} & -X- & \text{Pb}X_{4} \end{array}$	APbX_3_ ⇋ PbX_2_ + AX ⇋ Pb^0^ + AX_3_ In this view, no detail can be provided for p‐ or n‐ doping

In the chemical view (Table 1, right), bond breaking and re‐formation are described via the formation of AX_3_ species. AX_3_ is chemically stable and can oxidize metallic lead on the macroscale through a redox reaction (AX_3_ + Pb^0^ → Pb^2^^+^ + AX + 2X^−^) [143]. The I_3_^−^ species from AX_3_ can then react with the PbX_5_ octahedra. Note that writing such reactions does not imply formation of metallic Pb within the HaP lattice; even if Pb^0^ clusters form, the damage can remain reversible provided there is no loss of X_2_.

A final note concerns possible differences in the diffusion coefficients of interstitial halide (“X species”) defects under illumination, which may depend on light intensity. If these defects are long‐lived and act as inefficient traps [144], they may cycle between I_3_^−^ species, expected under p‐doping, and bridging I^−^ species, expected under putative n‐doping, by capturing and releasing charge. Upon returning to their original electronic configuration, there is a 50% probability of migration to a neighboring unit cell. This behavior is consistent with mechanisms used to describe defect/dopant diffusion in semiconductors, especially Si [145], such as the interstitial, substitution‐interstitial and Bourgoin–Corbett [146] ones, in which defect diffusion is assisted by changes in charge state.

If sufficiently fast, this process could enhance SH by reducing steady‐state defect densities and may explain the increased ion‐migration kinetics observed under illumination. Such an effect could scale supra‐linearly with light intensity, actively mitigating damage, provided the diffusion coefficient of interstitial species is comparable to or greater than that of vacancies. This framework may explain the results of Motti et al. [31], who observed higher steady‐state *PL* in MAPbI_3_ films illuminated from both sides than from one side only.

### 5.6 Acid‐Base Damage

In halide perovskites containing methylammonium (MA^+^) or formamidinium (FA^+^), a process analogous to vacancy–interstitial formation can occur without electron transfer: MA^+^ or FA^+^ can donate protons that bind to halides, producing methylamine (CH_3_NH_2_) or formamidine (HCNH_2_NH), denoted here as the neutral conjugate bases MA^0^ and FA^0^ (Figure 5a). Although we focus on MA^+^/MA^0^, the same arguments apply to FA^+^, which is less acidic and thus expected to deprotonate less readily. Illumination can promote deprotonation indirectly, as photoinduced heating, lattice dynamics, or local chemical changes can stabilize MA^0^, driving proton release. This constitutes a form of damage.

In early studies, proton‐related defects were not considered, because extrapolating from MA^+^ acidity in water predicts negligible deprotonation. However, in halide perovskites, the MA^0^ product might form a stabilizing methylamine–iodine complex, increasing the driving force for proton release. Similar stabilization may occur for FA^+^/FA^0^, though calculations are lacking. Relevant proton‐release reactions are summarized in Table 2 and Figure 5a. Importantly, protons can adopt different forms depending on doping: calculations indicate that they are typically bound to halides as H^+^ (Figure 5d), while in hypothetical highly n‐doped material (if they can be achieved) they would convert to trapped H_2_ (Figure 5e). The H^−^ state bound to Pb^2^^+^ is not stable [147, 148].

**TABLE 2:** Summary of the reactions involved in proton release in HaPs.

Bonds	Chemistry
N─H bond breaking CH_3_NH_2_ − H^+^ ⇋ CH_3_H_2_N: H^+^ H─X bond formation H^+^ + X_5_Pb − X − PbX_5_ ⇋ X_5_Pb − XH^+^ − PbX_5_	N─H bond breaking MA^+^PbX_3_ ⇋ [MA^0^PbX_3_]^−^ + H^+^ H─X bond formation H^+^ + [PbX_3_]^−^ ⇋ H^+^PbX_3_

If MA^+^ vacancies are present, they can be occupied by protons which can stabilise these vacancies with their positive charge (Figure 5g).

$$H^{+}+{\left[PbX_{3}\right]}^{-}⇋HPbX_{3}$$

In other words, a neighboring MA^+^ may deprotonate and its freed proton can fill a nearby $V_{{\mathrm{MA}}^{+}}$, remaining in place as MA^0^.

Though unverified by DFT, straightforward electrostatic considerations suggest this reaction is feasible (Figure 5b,c).

$$MA^{+}\mathrm{Pb}X_{3}+{\left[\mathrm{Pb}X_{3}\right]}^{-}⇋{\left[MA^{0}\mathrm{Pb}X_{3}\right]}^{-}+\mathrm{HPb}X_{3}$$

Notably, [*MA* ^0^ *PbX*_3_]^−^ (or simply MA^0^) can seemingly diffuse quickly, by propagating via proton capture and release from and to adjacent sites.

In oxide perovskites, proton diffusion occurs via hopping between oxygen atoms [149]. In halide perovskites, H^+^ “interstitials,” analogous to the Bourgoin–Corbett mechanism [146], may enable proton transfer between halides (I^−^, Br^−^) and nearby MA^+^ sites, altering the local electrostatic potential. While halide diffusion has been widely studied by DFT, far fewer works address proton diffusion [147, 150], and, to the best of our knowledge, only a single work [151] considers a Grotthuss‐type mechanism involving sequential proton capture and release by MA^+^ ions but it does not delve into its consequences.

Because SH kinetics may depend strongly on MA^0^ and H^+^ diffusion, preparation conditions can significantly affect SH rates. Water penetration can strongly modify proton mobility [73] and concentration, promote deprotonation, and thus influence damage/SH dynamics [42], as observed experimentally.

Finally, deprotonation is not a redox process: both electrons from the broken N─H bond remain on the nitrogen [152] and can be donated to undercoordinated Pb sites, passivating halide‐vacancy defects via N─Pb bond formation.

### 5.7 Link Between Redox and Acid‐Base Damage

If a unit cell with MA^0^ (which, missing a proton is negatively charged [MA_0_PbX_3_]^−^), also contains a positively charged halide vacancy, *V_X_*. MA^0^ can passivate *V_X_* by binding to a 5‐coordinated Pb site (Table 3, Figure 5c), forming a neutral defect.

**TABLE 3:** Summary of the methylamine passivation of halide vacancies in HaPs.

Bonds	Chemistry
CH_3_NH_2_( :) PbX_5_ ⇋ CH_3_NH_2_ − PbX_5_	CH_3_NH_2_ + Pb^2 +^ ⇋ CH_3_NH_2_Pb^2 +^

This favorable bond formation was identified by DFT calculations [21] (Figure 5f) and verified experimentally by reacting primary amines with PbI_2_ [153].

From a chemical perspective, MA^+^ acts as an acid that dissociates into a proton and the Lewis base MA^0^, which donates electrons to Pb^2^^+^, a Lewis acid. Outside an HaP, this would lead to further (reversible) methylamine deprotonation [153], but this reaction is specific to the perovskite environment.

This mechanism may explain photo‐brightening in some HaPs by converting two charged defects into a neutral complex, linking redox and acid–base chemistry and influencing defect creation and healing kinetics. We discuss below how this (*V_I_* + MA^0^) complex affects *PL* and power conversion efficiency under illumination.

**FIGURE 6:**
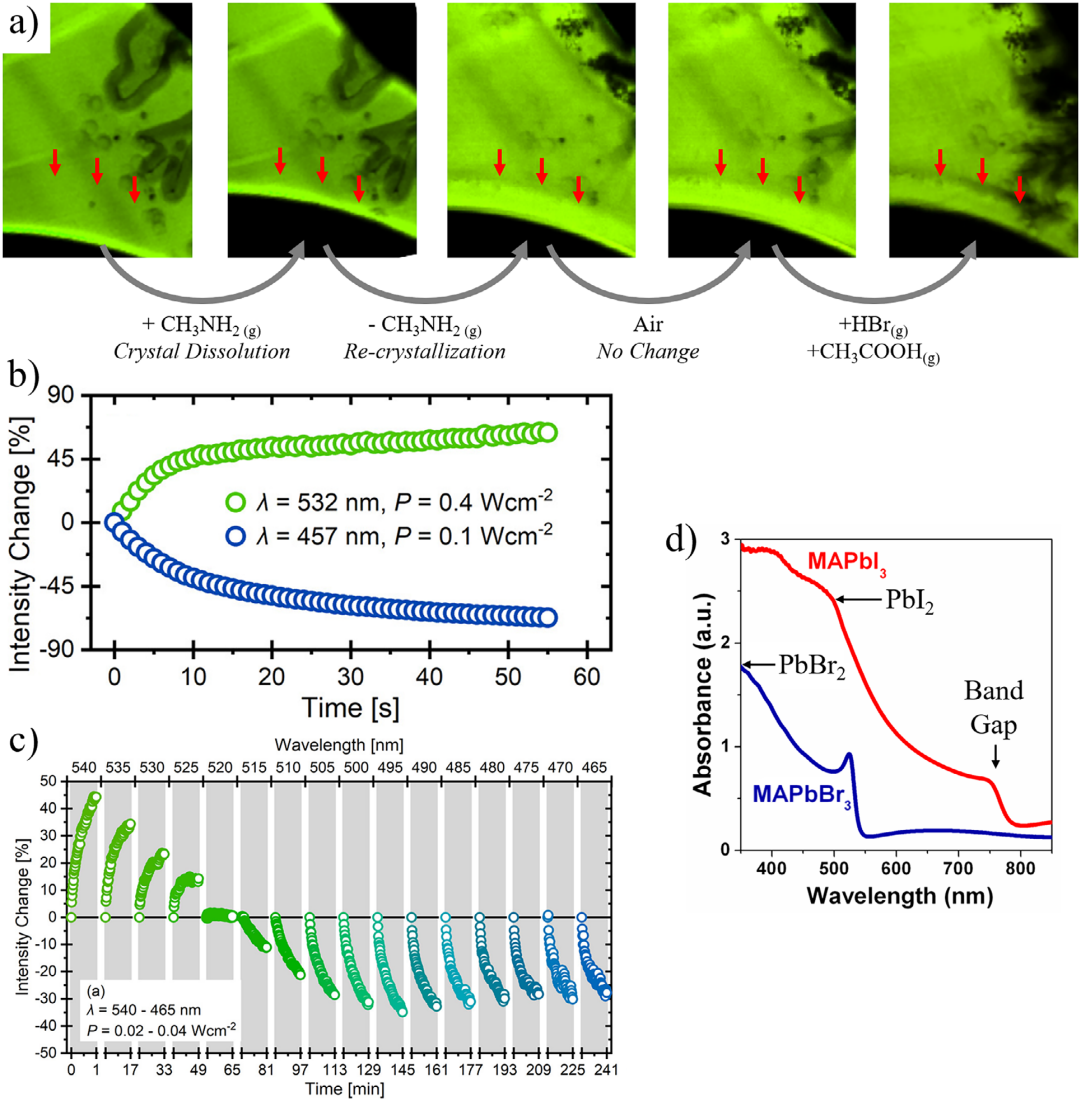
(a) Two‐photon confocal microscopy *PL* images from inside a MAPbBr_3_ crystal with the edge shown at the bottom, where dissolution (and recrystallization) occurs following exposure to methylamine and then to an acidic environment (red arrows show the same region across the five images, shown from left to right). *Leftmost*: The pristine crystal emits uniform *PL*, indicating good optical quality. *Left*: Upon exposure to methylamine gas, the crystal begins to dissolve. *PL* disappears in the bottom region, consistent with loss of material. *Middle*: After removing the methylamine atmosphere, the HaP recrystallizes (additional bright region), with no apparent boundary between the original and reformed material. The *PL* intensity in the recrystallized region is higher than in the original crystal, consistent with defect passivation typically observed after methylamine exposure and recrystallization. *Right*: Even after prolonged exposure to ambient air, the enhanced *PL* persists. This can be if methylamine remains trapped in the lattice and does not fully evaporate, preserving the improved optical quality. *Rightmost*: When the recrystallized region is exposed to an acidic environment, a new driving force enters the system. Protons likely diffuse into the crystal and react with the methylamine that passivated defects. As a result, the *PL* in the previously recrystallized region decreases and becomes similar to that of the untreated part of the crystal, suggesting that the defect passivation has been reversed. Data were acquired from the bulk region, positioned 100 µm below the top surface of a MAPbBr_3_ single crystal, across an observed area of 400 × 540 µm^2^. The images were obtained by 2‐photon confocal *PL* microscopy. The dark features present in the right top and middle parts of the images are shadows projected by partially delaminated tape on the crystal's top surface, used to protect this surface from the ambient. As the relevant parts of these images are at the bottom, they are not affected by those features. This experiment demonstrates that proton chemistry plays a fundamental role in at least one process that is responsible for *PL* enhancement in halide perovskites. (b) Evolution of the *PL* of a MAPbI_3_ crystal exposed to 532 and 457 nm LED light; (c) evolution of the same sample exposed to different wavelengths from a monochromator. (b,c) are from ref. [51]. (d) The absorption spectra of MAPbBr_3_ (Blue) and MAPbI_3_ (Red) adapted from ref. [161] showing the transitions above the band gap. For MAPbBr_3_ a similar transition is found around 350 nm (from ref. [157]).

To support the role of methylamine or formamidine in passivation, we present external evidence showing that defect passivation requires different energies than defect formation. Figure 6a demonstrates the central role of acid–base chemistry. Exposing a MAPbBr_3_ single crystal to methylamine gas causes dissolution and recrystallization, leading to increased *PL*, consistent with passivation of bromide vacancies [154]. Exposure to an acidic environment (HBr in CH_3_COOH) reverses this effect, restoring the original PL. Thus, a proton‐poor environment enhances *PL* and, by extension, efficiency (*η*) in halide perovskites.

Those results strongly suggest that methylamine effectively passivates defects that are detrimental for optoelectronic performance, with important implications for identifying which defects truly matter, as discussed in the final section.

A second, often overlooked factor, closely linked to redox and acid–base damage, is the wavelength (energy) dependence of light‐induced effects. Few studies address this issue, due to experimental challenges. Those that do indicate a transition around 2.4–2.5 eV (∼520 nm) in MAPbI_3_ and related perovskites [30, 51, 56, 89, 155]. Below this threshold, illumination typically causes photobrightening, while higher‐energy photons reduce PL.

These results imply that lower‐energy photons generate passivating defects, while higher‐energy photons create more harmful ones. Figure 6b shows *PL* evolution in MAPbI_3_ under 532 nm (photobrightening) versus 457 nm (photodamage) excitation, and wavelength scans (Figure 6c) reveal a clear transition near 520 nm [51]. This threshold may reflect weak PbI_2_ absorption or, more likely, higher‐energy electronic transitions [156] above the bandgap (Figure 6d). High‐energy absorption may locally excite PbI_6_ octahedra, triggering photochemical reactions. Although the link between carrier thermalization and bond breaking is not fully understood, Pb‐I bond breaking likely provides an additional relaxation pathway for carriers excited above ∼2.4 eV.

These findings are central to understanding halide perovskite degradation and controlling defect chemistry. Similar wavelength‐dependent behavior is expected in MAPbBr_3_, shifted to ∼3.55 eV (350 nm) due to its higher‐energy transition (around 3.55 eV (350 nm)) [157], though this remains to be confirmed experimentally. Comparable passivation processes should also apply to formamidinium‐based perovskites, with analogous reactions involving formamidine (which can be obtained in the gas [158] phase).

### 5.8 Irreversible Reactions

Up to this point, the discussion has focused on reversible chemical processes that establish dynamic equilibria or steady states, allowing the perovskite to recover its original composition provided all species remain confined. However, a fundamentally different pathway exists in which volatile products and/or secondary reaction products cannot realistically recombine. Under operationally relevant light/thermal stress, MAPbI_3_ has been reported to decompose through a reversible channel releasing CH_3_NH_2_ + HI, and a more detrimental/irreversible channel producing NH_3_ + CH_3_I [87].

At elevated temperatures, the equilibrium CH_3_NH_3_I ⇌ CH_3_I + NH_3_ can be established; however, the reverse reaction is not fully selective/reversible in practice because CH_3_I is reactive and can be consumed by secondary reactions, depleting the precursors needed to re‐form methylammonium iodide [88, 159]. Consistent with this, photodegradation experiments detect NH_3_ and CH_3_I among the released volatiles and indicate formation of additional/complex organic products under illumination. In particular, in the presence of CH_3_I, methylamine may undergo further alkylation to form di‐ and tri‐methylamine [160]; however, since these likely form only locally and there is no excess CH_3_I overall, methylammonium iodide is expected to remain the main product.

In formamidinium‐based perovskites (FA^+^), analogous irreversible sinks have been identified. Thermal decomposition of FAPbI_3_ and of FAI has been reported to generate HCN together with condensed N‐rich organic products, frequently identified with sym‐triazine (s‐triazine), alongside NH_4_I (and at higher temperature NH_3_/HI from subsequent NH_4_I decomposition) [162]. The condensed product composition can depend on conditions and interfaces: while several TG‐MS/thermal studies report s‐triazine + HCN, other work has found 2‐aminomalononitrile (rather than triazine) as the dominant condensed organic product in FAPbI_3_ decomposition, still accompanied by HCN [163]. Interfacial contact (e.g., with NiO_x_ or TiO_2_) can lower the onset temperature for FAI and FAPbI_3_ decomposition and alter the product distribution, making FA^+^‐related irreversibility strongly stack‐dependent [90]. Finally, FAI can decompose during vacuum evaporation into HCN and s‐triazine, showing that these sinks can form even in processing‐relevant environments [164].

Electrical bias can further introduce irreversible reaction pathways by enabling electron injection and facilitating proton reduction. Under such conditions, when substantial amounts of CH_3_I are present, H_2_ evolution and formation of diiodomethane (CH_2_I_2_) have been observed [164]. Because H_2_ readily escapes from any realistic confinement, these pathways render degradation definitively irreversible. Thus, CH_3_I + NH_3_ formation constitutes a true microscopic sink, marking the boundary between reversible self‐healing chemistry and irreversible degradation. Finally, it may be of interest to investigate whether iodine–amine adduct formation and N‐iodoamine species in non‐aqueous media (e.g., CH_3_NHI/CH_3_NI_2_) [165, 166, 167] could enable iodine‐mediated N–N coupling (“azine/hydrazine‐type”) chemistry under iodine‐rich conditions, as demonstrated for amines in related systems. Even though direct experimental evidence for this degradation pathway during perovskite degradation has not yet been reported, higher‐mass fragments of this kind may not be routinely considered when interpreting mass‐spectrometry data, because such signals could plausibly be attributed to contamination or background when they fall outside the set of fragments typically expected or searched for.

### 5.9 *PL* as a Proxy of the Defect Concentration

In HaPs, *PL* offers a direct and practical handle on monitoring defect dynamics under illumination and during recovery. In regimes where defect‐assisted recombination dominates, *PL* can be approximated as inversely proportional to the concentration of active non‐radiative centers. Assuming low‐injection conditions and a single dominant defect type, for the time‐dependent *PL*(*t*) one obtains (Table 4):

**TABLE 4:** Expected *PL*, normalized *PL*, and normalized defect density (normalization to the pre‐damage value) during photo‐damage and SH in HaPs.

Time‐dependent *PL*	$PL\left(t\right)\approx \;\frac{G}{N_{eq}+\Delta N\left(t\right)}$
Normalized *PL*	$\;\frac{PL(t)}{PL_{0}}=\frac{N_{eq}}{N_{eq}+\Delta N\left(t\right)}$
Photo‐generated defects	$\;\frac{\Delta N(t)}{N_{eq}}=\frac{PL_{0}}{PL(t)}\;-1$

Here, *N_eq_* is the equilibrium defect concentration and Δ*N*(*t*) the excess (or deficit) induced by light exposure and *PL*_0_ is the *PL* before damage (=*G*/*N*_eq_; where *G* is the free carrier generation rate and *N*_eq_ the equilibrium concentration of the defects with the strongest effect on *PL*). This relationship allows one to estimate the relative defect density at any point in time without specifying the exact defect identity, with increasing (decreasing) *PL* implying defect passivation/ annihilation (accumulation/activation).

In general, a *PL* decrease is attributed to the increase of a specific type of recombination‐active defect. However, in reality, the recombination rate that controls *PL* is not governed by a single defect but rather by the collective contribution of many. Each defect type, halide vacancies and interstitials, proton vacancies, methylamine‐related species, and others, has its own recombination efficiency. The net impact on *PL* results from a weighted average of all these contributions, depending on both their density and individual rate constants. Static defect calculations suggest that interstitial defects, that is, $X_{3}^{-}$ or 2*X* ^−^ (as in Figure 4c,d) impact the efficiency of *PL* and solar cell performance in perovskites more than vacancies, *V_X_*. The reason is that the charge/discharge levels of the latter are found, in such calculations, to lie in the bands, outside the bandgap [141, 168]. However, this hypothesis requires experimental validation, which could be achieved by controlling the chemical potential of halides during material synthesis. Moreover, in a realistic visualization of a dynamic lattice of perovskites, *V_X_* charge/discharge levels even outside the bandgap in the 0 K structure, can become in‐band‐gap energy levels because the lattice (and consequently energy) fluctuations make 0 K calculations not relevant near RT [137]. Additionally, it has been proposed that proton vacancies in methylammonium‐containing perovskites may serve, under some conditions, as even deeper traps than halide interstitials [152].

We propose a model with two chemically distinct, photo‐generated defect species: one that reduces *PL* and another that does not but can eliminate or passivate the first. For illustration, we assume iodine vacancies dominate *PL* loss; the argument holds equally if interstitials are responsible, provided a passivating species exists. In any case, both vacancies and interstitials can participate in SH, and their density jointly can determine healing rates. Within this framework, proton‐related species (H^+^ vacancies or MA^0^) do not directly affect *PL* but regulate it by passivating iodine vacancies (Figure 5b,c,f). Thus, *PL*‐active and passivating defects are distinct: one controls recombination, the other its abundance.

Importantly, the model is symmetric: if proton vacancies were the true recombination centers and halide chemistry the passivating mechanism, the reasoning would be unchanged. What matters is that illumination generates two chemically distinct defect species, one that reduces *PL* and another that does not but can passivate the first. This two‐component view, separating recombination centers from chemical regulators, provides a robust framework for interpreting *PL* behavior in a defect landscape that evolves with time, composition, and environment.

This framework enables experimental data to be used not only as qualitative stability markers but also to extract recombination constants and defect densities, advancing a predictive understanding of SH in HaPs based on defect kinetics. Quantitative analysis and literature data will be addressed in a follow‐up publication, explaining why *PL* can increase, decrease, and then increase again, or behave oppositely, in similar samples. Such trends depend strongly on the initial defect state and its underlying chemistry. We will also show how external species, such as water, alter diffusion coefficients and recombination rates of mobile defects.

### 5.10 SH Rate ↔ Defect Concentration

As a final remark, we note that although we have shown that SH is generally beneficial, it often raises doubts in the community. In particular: (1) if SH relies on weak bonds, does its presence signal an intrinsically unstable material that should be avoided? (2) do SH kinetics control the defect concentration, and if so, how?

Regarding (1), weak bonding is indeed required for SH, but it does not imply macroscopic instability. A simple *reductio ad absurdum* is liquid water: hydrogen bonds are weak and continuously broken and re‐formed, yet the liquid remains stable. Weak bonds are therefore a necessary but not sufficient condition for SH and do not, by themselves, imply material fragility.

Regarding (2), the answer is yes. If SH kinetics are reasonably fast, the perovskite relaxes toward its thermodynamically dictated defect concentration, which is low and largely independent of sample history (apart from morphology and surface area, because of surface defects). Under illumination, defects are continuously generated, but they are also healed, leading to a steady‐state defect population. The faster the SH kinetics, the lower this steady‐state defect concentration must be. In the liquid analogy, a low‐viscosity liquid (fast SH) returns to equilibrium more rapidly after a perturbation than a viscous one (slow SH). Thus, fast SH naturally enforces a low defect density.

This provides a rationale for why the most efficient perovskite formulations (double‐ and triple‐cation systems) are FA‐rich: FA, which is known to show fast SH kinetics, forms the main lattice. MA, beyond facilitating the control of crystallization and the formation of larger grains (which improves charge transport and stability) [169, 170, 171, 172], passivates additional defects via complex formation (Figure 5f). Cs, even though making up to 5 mol% or even less of the A cations, acts as a stabilizing degradation suppressor by reducing organic loss and forming a Cs‐rich surface layer on the grains. The latter effect is analogous to what makes steel corrosion‐resistant: there the alloying additions can be very low, for example, ∼1–3 wt.% Ni in Ni‐advanced weathering steels or a few wt.% Mo in Mo‐bearing stainless steels) [173, 174, 175] can disproportionately enhance durability by enriching protective near‐surface rust/oxide layers [176] and thereby slowing corrosion).

## 6 Conclusion

This review set out to answer a deceptively simple question: how do lead‐halide perovskites self‐heal from damage? By assembling and critically analysing experimental observations across illumination, electrical bias, mechanical stress, temperature, and radiation exposure, we show that self‐healing (SH) in APbX_3_ perovskites is neither incidental nor mysterious, but a direct consequence of their special chemical and structural characteristics.

A central conclusion is that degradation and recovery are inseparable manifestations of the same underlying chemistry. Damage corresponds to bond cleavage within the Pb─X framework and, in hybrid organic–inorganic perovskites, to acid–base reactions involving A‐site organic cations. Recovery proceeds through the reverse processes: re‐bonding, redox equilibration, and defect passivation mediated by mobile species such as halides, protons, and neutral amines. In this sense, SH is not an extrinsic repair mechanism but an intrinsic, thermodynamically driven response of a soft, dynamically bonded lattice.

Our analysis shows that “defect tolerance” alone cannot account for the observed behavior. Instead, the apparent stability of halide perovskites is governed primarily by kinetics: the balance between defect generation and annihilation, set by diffusion coefficients that span many orders of magnitude. As a result, devices can operate in dynamic steady states, cycling between degraded and healed configurations over times ranging from seconds to days. Composition, lattice strain, nanoscale confinement, and environmental conditions strongly modulate these kinetics, explaining the wide variability reported in the literature.

A key conceptual advance presented here is the separation between electronic charge recombination‐active defects and chemically distinct species that regulate their population. This two‐component framework provides a consistent interpretation of photodarkening, photobrightening, light soaking, and complex *PL* transients, while remaining agnostic about the precise microscopic identity of the dominant recombination centers. It also clarifies the central role of acid–base chemistry, particularly proton release and methylamine or formamidine formation, in linking redox processes to defect passivation.

Self‐healing has important practical implications. It explains the unexpectedly high radiation tolerance of halide perovskites, their partial resilience under prolonged operation, and the critical importance of hermetic encapsulation to prevent irreversible mass loss or parasitic reactions. More broadly, it reframes stability not as a static materials property but as a dynamic one that can be engineered by controlling chemistry, structure, and kinetics.

Altogether, the evidence supports a view of halide perovskites as thermodynamically controlled, adaptive materials whose functionality emerges from the dynamic interplay of damage and repair. Recognizing and exploiting this self‐healing capability transforms it from a serendipitous curiosity into a predictive design principle, essential for advancing HaP technologies in photovoltaics, light emission, and radiation detection.

## Conflicts of Interest

The authors declare no conflicts of interest.

## Data Availability

The authors have nothing to report.
